# Stroke in people living with HIV and 6-month outcomes in Northwestern Tanzania

**DOI:** 10.1016/j.neuros.2026.100063

**Published:** 2026-07-08

**Authors:** Lilian Andrew Mwamba, Emmanuel M. Bukelebe, Innocent Kitandu Paul, Joshua Ngimbwa, Matilda K. Basinda, Benjamin Andrew, Amon B. Paul, Akili Mawazo, Dorice Lucas, Ladius Rudovick, Bahati Wajanga, Samuel Kalluvya, Paul Olowoyo, Robert Peck, Sarah Shali Matuja

**Affiliations:** a Department of Neurology, First Affiliated Hospital of Jinzhou Medical University, Liaoning, China; b Department of Internal Medicine, Catholic University of Health and Allied Sciences-Weill Bugando School of Medicine, Mwanza, Tanzania; c Department of Internal Medicine, Aga Khan University, Dar-es-salaam, Tanzania; d Department of Internal Medicine, Bugando Medical Centre, Mwanza, Tanzania; e Department of Microbiology and Immunology, Muhimbili University of Health and Allied Sciences, Dar es Salaam, Tanzania; f Department of Medicine, Federal Teaching Hospital Ido-Ekiti, Afe Babalola University, Ado-Ekiti 360211, Nigeria; g Center for Global Health, Department of Internal Medicine, Weill Cornell Medicine, New York, NY, USA

**Keywords:** Stroke, HIV, HIV-associated stroke, 6-Month outcomes, Tanzania

## Abstract

**Introduction:**

Stroke in HIV presents with distinct characteristics and varying post-stroke recovery patterns, particularly in the antiretroviral therapy (ART) era. We aimed to evaluate the current trends, characteristics, and 6-month outcomes among people living with HIV (PLWH) compared to people without HIV (PWOH).

**Methods:**

This prospective cohort study included adults with stroke registered in the Lake Zone Stroke Registry at Bugando Medical Center between November 2023 and March 2025. Baseline characteristics, stroke severity, and outcomes were assessed using the National Institutes of Health Stroke Scale (NIHSS) and the modified Rankin Scale (mRS), respectively. The Kaplan–Meier analysis was used to describe survival, and the Cox regression model to determine predictors of mortality.

**Results:**

We analyzed 295 adults, with an HIV proportion of 8.8% (95% CI: 5.9%−12.7%) (26/295). The median age for the overall study cohort was 66 years (IQR 53.0–77.0), and 55.9% (165/295) were males. Among PLWH, the median age was 53.5 years (IQR 44.3–67.0), females were 61.5% (16/26), and the majority had hypertension, 57.6% (15/26). The median CD4 count was 518 cells/μl (IQR 151.5–571), and 84.6% (22/26) were naïve to ART. PLWH experienced a longer hospital stay, 5 IQR (2–7) days. Both groups experienced unfavorable functional outcomes (mRS 3–6), 84.7% (250/295), and a mortality rate of 38.6% (114/295) at 6 months. In the multivariable analysis, dyspnea, presence of leukocytosis, and severe stroke were independent predictors of mortality.

**Conclusion:**

Although 6-month outcomes were comparable between both groups, targeted strategies are needed to address healthcare disparities and reduce the additional burden of stroke in PLWH.

## Introduction

Stroke remains the second leading cause of death, accounting for 7 million deaths, and the third leading cause of death and disability adjusted life years (DALYs) combined, responsible for over 160 million DALYs [1]. There is an increased stroke burden in low- and middle-income countries (LMICs) contributing to 87.2% of deaths and 89.4% of DALYs of the global stroke burden [2,1]. Sub-Saharan Africa (SSA) bears an increased prevalence of stroke and other non-communicable Diseases (NCDs) because of improved life expectancy due to advancements in early diagnosis, treatment, and control of HIV/AIDS and other infectious diseases [3–5]. A large community-based study in Tanzania demonstrated an age-standardized stroke incidence rate of about 316 per 100,000 population, higher than observed in most studies in high-income countries (HIC) [6].

Stroke has often been discussed in the context of HIV infection, with several biological mechanisms proposed to explain how HIV may influence cerebrovascular disease, including vasculopathy, cardio-embolism, coagulopathy, and chronic inflammation [7–10]. While much of the literature has focused on HIV as a potential risk factor for the occurrence of stroke, far less attention has been given to how HIV status may influence the clinical presentation, severity, and outcomes of stroke once it occurs.

In SSA, where both HIV and stroke are highly prevalent, emerging evidence suggests that stroke in people living with HIV (PLWH) may present with distinct characteristics, including younger age at onset [11–13], differences in stroke subtypes on imaging, and varying post-stroke recovery patterns compared to people without HIV (PWOH), particularly in the era of expanding antiretroviral therapy (ART) access [7,14,15]. However, most available data are derived from high-income settings, and there is limited contemporary evidence from SSA examining how HIV status relates to stroke phenotype and post-stroke outcomes.

In Tanzania, a hospital-based cross-sectional study conducted more than a decade ago demonstrated a notable HIV prevalence of 20.9% among individuals with stroke, with a lack of data addressing the outcomes of these patients in this population [16]. Given the widespread use of ART and the lack of current data, we aimed to investigate the recent proportion of HIV among adults admitted with stroke and to examine how HIV status relates to stroke characteristics and 6-month outcomes at a tertiary teaching hospital in Northwestern Tanzania. This study focuses on understanding HIV as an exposure influencing post-stroke presentation and outcomes rather than stroke occurrence itself.

## Methods

### Study setting and population

This cohort’s study participants were admitted at Bugando Medical Center (BMC), a large tertiary and teaching hospital, and were registered in the Lake Zone Stroke Registry (LZS) in Northwestern Tanzania [17]. We included all adults (≥ 18 years) who met the World Health Organization (WHO) clinical definition for first/recurrent stroke, defined as a rapidly developing clinical sign of focal (or global) disturbance of cerebral function lasting more than 24 hours or leading to death, with no apparent cause other than vascular origin [18]. BMC serves as a catchment area of more than 15 million individuals [19]. It is the main referral hospital for people with strokes, particularly those who reside along the shores of Lake Victoria. Within the Neurology Unit, stroke admissions comprise approximately 80% of all admissions for neurological disorders, with 250–300 stroke admissions annually [17]. This proportion refers specifically to admissions within the Neurology Unit and does not represent the proportion of stroke among all admissions to the Department of Internal Medicine. BMC has recently established a dedicated 8-bed stroke unit (August 2024) to enhance acute stroke care and treatment [20]. During most of the study period, however, stroke patients were managed within the general 150-bed adult medical wards. Stroke management primarily focuses on acute medical stabilization and secondary prevention. Although physiotherapy services are available for both inpatients and outpatients, structured multidisciplinary inpatient stroke rehabilitation remains limited. Dedicated speech and language therapy and comprehensive inpatient rehabilitation programs are not routinely available. Consequently, once clinically stable, most patients are discharged home and continue recovery under family support, with outpatient physiotherapy when accessible.

### Data collection

Consecutive stroke patients were recruited and registered using a digital web-based software, REDCap, between November 2023 and March 2025, and followed up until September 2025. Baseline information collected included: age, sex, marital status, occupation, health insurance coverage, referral status, residency, and education level. Urban residences were primarily defined by administrative designation, size, density, and the predominance of non-agricultural activities, while rural residences were referred to areas outside these urban parameters, typically characterized by agricultural livelihoods and lower population density. Semi-urban residences were defined as regions that exhibit characteristics of both rural and urban residences, typically featuring moderate population density, basic infrastructure development, and a blend of agriculture and small-scale commerce [21,22].

Previous comorbidities such as HIV infection, hypertension, diabetes mellitus, smoking, alcohol use, previous stroke, cardiac diseases, and dyslipidemia were recorded. Hypertension was defined as systolic blood pressure (SBP) ≥140 mmHg and/or diastolic blood pressure (DBP) ≥90 mmHg or previous/current use of anti-hypertensive medications [23,24]. Diabetes mellitus diagnosis was defined as a fasting blood glucose (FBG) of ≥ 7.0 mmol/L (126 mg/dL), HbA1C ≥ 6.5 %, random blood glucose (RBG) of ≥ 11.1 mmol/L with classical symptoms or previous/current use of hypoglycemic medications/insulin [25]. Dyspnea was defined as a subjective symptom of breathlessness or chest tightness reported by the patient or caregiver at presentation after an acute stroke [26,27]. This was recorded as a presenting symptom and did not represent a confirmed clinical diagnosis, as it could reflect underlying respiratory, cardiac, or stroke-related causes [28].

All adults underwent neuro-imaging using a non-contrast computed tomography scan, acquired on a 128-slice CT scanner (Siemens Somatom Perspective, Siemens Healthcare GmbH, Germany), and findings were classified as ischemic, hemorrhagic, normal, and ischemic with hemorrhagic transformation by a radiologist. Ischemic stroke diagnosis was based on imaging findings consistent with infarction and corresponding clinical presentation. Small vessel (lacunar) infarcts were classified based on characteristic clinical syndromes and compatible CT findings when present. Magnetic resonance imaging (MRI) was performed selectively when clinically indicated, but was not routinely obtained for all patients.

Patients with confirmed ischemic stroke on imaging (including those with hemorrhagic transformation) were further classified according to the Trial of ORG 10172 in Acute Stroke Treatment (TOAST) criteria [29] when sufficient etiologic data were available. TOAST categorization was applied only in patients who had completed a predefined minimum etiologic evaluation as part of routine clinical care. This evaluation included 12-lead electrocardiography (ECG), transthoracic echocardiography (TTE), carotid Doppler ultrasound, brain CT and/or MRI, and CT angiography when clinically indicated. Patients were assigned to one of the standard TOAST categories when criteria were met. “Undetermined etiology” was used when the minimum evaluation was completed, but no single cause was identified, or when more than one potential cause was present. Advanced investigations required to reliably identify less common etiologies (e.g., prolonged cardiac rhythm monitoring, thrombophilia testing, autoimmune evaluation, or specialized vascular imaging) were not routinely available. Therefore, TOAST classification was not possible in all cases, and patients without sufficient etiologic evaluation were recorded separately as “unclassified due to insufficient etiologic data.” Hemorrhagic stroke was classified according to hematoma location.

Stroke severity was assessed using the National Institutes of Health Stroke Scale (NIHSS) on admission [18], with scores ranging from 1 to 42, categorized as minor (1–4), moderate (5–15), moderate to severe (16–20), and severe (21–42) scores, based on previously published categorizations used in clinical stroke assessment and research [30–33].

Laboratory investigations were completed on admission, including complete blood counts (CBC), total cholesterol levels, low-density lipoprotein (LDL), and random blood glucose (RBG). Leukocytosis was defined as a leukocyte count > 11.1 × 10^9^ /L; total cholesterol > 240 mg/dL, and LDL cholesterol > 160 mg/dL were considered elevated. HIV screening and diagnostic tests were performed using a rapid diagnostic test designed to detect HIV antibodies/and or antigens. The screening test utilized was the Abbott Bioline HIV1/2 3.0, which requires a small volume of whole blood collected via a finger prick. For confirmation of reactive screening results, the Uni-Gold HIV (Trinity Biotech Manufacturing Ltd) was employed as recommended by the Tanzanian National Guideline for HIV Testing [34].

Functional outcomes were assessed using the modified Rankin Scale (mRS; scores 0–6) by trained medical doctors from the neurology unit using a structured interview approach to promote consistency and reliability. All assessors were familiar with standardized mRS scoring procedures. Pre-morbid functional status was assessed retrospectively at the time of admission. Admission and discharge functional status were assessed in person during hospitalization. Follow-up functional outcomes at 1, 3, and 6 months were assessed via structured telephone interviews with the patient or caregiver.

For analysis, mRS scores were dichotomized into favorable outcome (0–2) and unfavorable outcome (3–6) based on established conventions in stroke outcome research [35]. We acknowledge that dichotomization may reduce granularity within outcome categories. The date of death was recorded, and the time-to-event was calculated as the interval between stroke symptom onset and the date of death or last follow-up.

### Statistical analysis

All data were cleaned, entered, processed in Microsoft Excel, and analyzed using IBM SPSS version 25. The distribution of numerical variables was done by visual inspection of histograms and Q-Q plots to assess for normality. Normally distributed variables were summarized as mean and standard deviation (SD), whereas non-normally distributed variables were summarized as median with interquartile range (IQR). Comparisons between PLWH and PWOH were computed using the Chi-square test for categorical variables, the independent sample t-test for normally distributed continuous variables, and the Mann-Whitney U test for non-normally distributed variables. Kaplan-Meier analysis was used to estimate survival probabilities over time, and differences in survival between groups were assessed using the log-rank test. The associations between independent predictors associated with 6-month mortality were analyzed using the Cox regression hazard model. The multivariable Cox regression model was developed using a combination of literature-based and data-driven approaches. Variables identified from previous studies as clinically relevant predictors of stroke mortality, including age, sex, referral status, health insurance status, and HIV status, were included as potential confounders [12,36–38]. In addition, variables with a p-value < 0.20 in the univariable analysis (dyspnea, CT findings, admission NIHSS category, and leukocyte count) were entered into the multivariable model. Missing data were assessed for all variables, and a complete-case analysis was performed, excluding participants with missing data on any predictor or outcome variable. Multicollinearity among the independent variables was assessed using tolerance values and variance inflation factors (VIF). Hazard ratios (HR), 95% confidence intervals (CI), and corresponding p-values were obtained from the models adjusted for potential confounders. The significance level was set at a p-value of < 0.05.

## Results

### Baseline characteristics

**Fig. 1.**
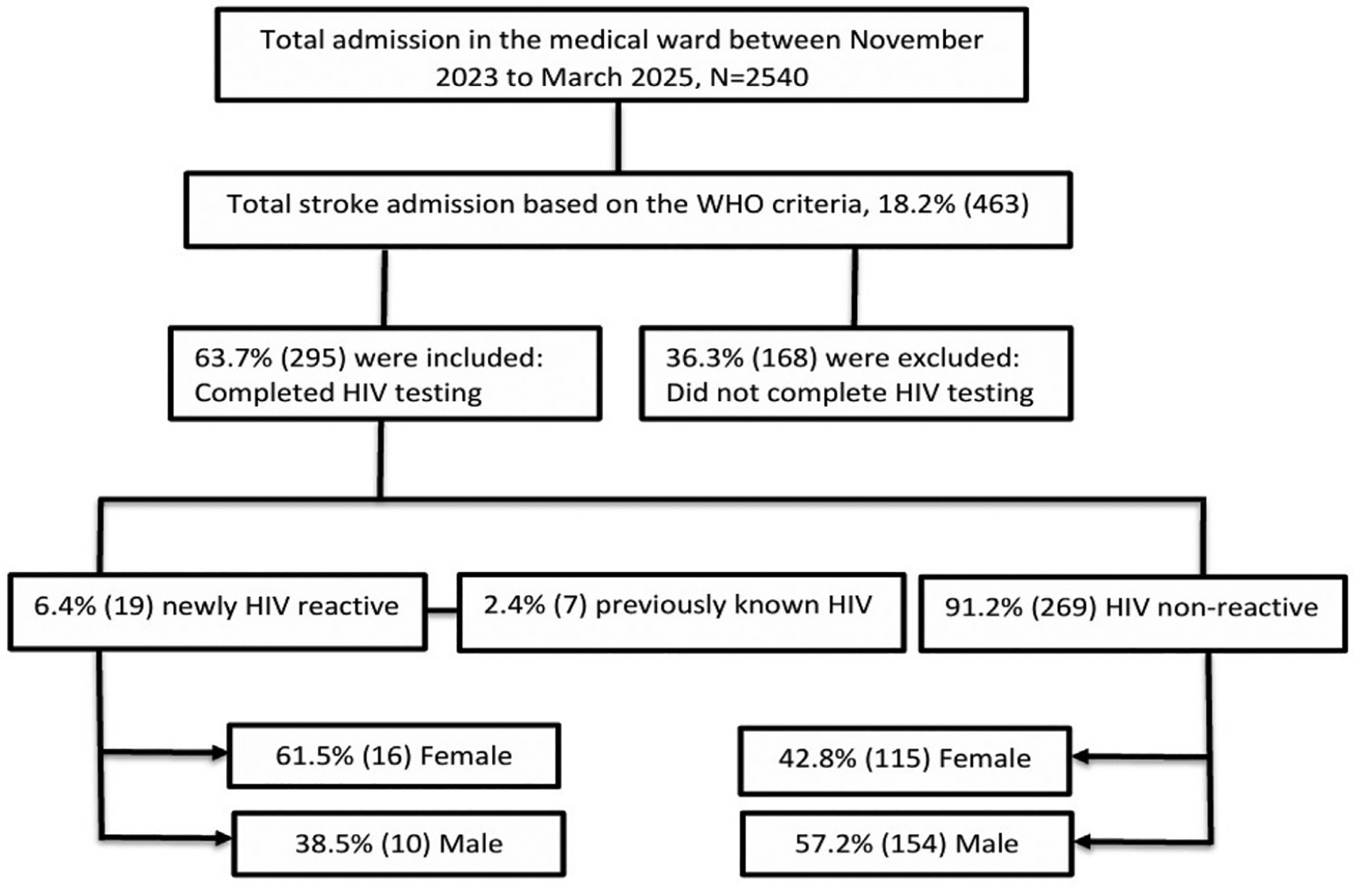
Flow diagram of the selection process.

Between November 2023 and March 2025, there were 2,540 admissions to the Department of Internal Medicine, including the Neurology, Gastroenterology, Infectious Diseases, Cardiovascular, Renal, Intensive Care Unit (ICU), and General Medical units. Of these admissions, 18.2% (463/2540) met the WHO clinical definition of stroke. We excluded 36.3% (168/463) adults with stroke for the following reasons: 2.4% (11/463) had stroke mimics, 24.6% (114/463) did not complete HIV testing during the hospital admission, and 9.3% (43/463) died before HIV testing. We therefore included 63.7% (295/463) in the final analysis, Fig. 1.

A comparison between included and excluded patients is presented in Supplementary Table S1. Compared to the included group, excluded patients were younger with a median age of 63.0 years (IQR 54.3–74.3) vs 65.0 years (IQR 52.0–76.0) (p=0.001). They were more likely to reside in urban areas, 50.6% (85/168) vs 30.5% (90/295), (p=0.001), had higher educational attainment, 22.6% (38/168) vs 11.9% (35/295), (p=0.001) and frequent previous stroke 15.5% (26/168) vs 9.2% (27/295), (p=0.043).

Additionally, premorbid functional status differed significantly between groups. The excluded group had a higher proportion of patients with moderate disability (mRS 3: 26.2% [44/168] vs 8.1% [24/295], p = 0.001) and moderate to severe disability (mRS 4: 17.3% [29/168] vs 6.1% [18/295], p = 0.001) compared with the included group. In contrast, admission stroke severity based on NIHSS categories did not differ significantly between groups (severe stroke [NIHSS 21–42]: 33.9% [57/168] vs 38.6% [114/295], p = 0.087). Similarly, in-hospital mortality (mRS 6) was not statistically different (25.6% [43/168] vs 31.9% [94/295], p = 0.060), Supplementary Table S1.

The overall proportion of HIV was 8.8% (95% CI: 5.9%−12.7%) (26/295), where 6.4% (19/295) were newly diagnosed, and 2.4% (7/295) were previously known with HIV. A total of 91.2% (269/295) were non-reactive to HIV, Fig. 1. The median age for the study cohort was 66.0 years (IQR 53.0–77.0), and 55.9% (165/295) were males. Most patients were married 98.6%, (291/295). A total of 38.6% (114/295) resided in semi-urban areas, and 60.7% (179/295) lacked health insurance coverage. Primary-level education was reported by 41.0% (121/295) of patients. Ischemic stroke accounted for 49.8% (147/295), and 42.4% (125/295) had hemorrhagic stroke, with hypertension being the most prevalent risk factor, 69.5 % (205/295). Furthermore, the mean (SD) NIHSS score on admission was 19.8 ± 5.8; of the 295 patients, 44.7% (132/295) had moderate to severe stroke (NIHSS scores 16–20), and 38.6% (114/295) had severe stroke, Table 1.

Among PLWH, the majority were females, 61.5% (16/26), and 57.7% (15/26) had hemorrhagic stroke. The median CD4 count was 518 cells/μl IQR (151.5–571) cells/μl, and among known HIV adults with stroke, 15.4% (4/26) were on regular ART. Baseline characteristics were similar between PLWH and PWOH except, PLWH were significantly younger, 53.5 years (IQR 44.3–67.0) vs 66.0 years (IQR 54.0–78.0), p = 0.001, more likely to have attained primary-level education, 50% (13/26) vs 40.1% (108/269), (p=0.002), had lower median LDL levels 2.6 mmol/l (IQR 1.5–3.7) vs 3.4 mmol/l (IQR 2.7–4.2), (p=0.037) and were more likely to have functional disabilities prior to index stroke 76.6% (206/269) vs 57.6% (15/26), (p=0.006), compared to PWOH, Table 1.

### CT findings

Baseline characteristics by imaging findings showed that PLWH who experienced ischemic stroke were younger, 59.0 years (IQR 49.5–70.0) vs 70.0 years (IQR 62.0–80.0) in PWOH, p = 0.016, Table 2. Similarly, PLWH and hemorrhagic stroke were younger, 49.0 years (IQR 42.0–64.0) vs 59.0 years (IQR 48.0–69.9) in PWOH, but the difference did not reach statistical significance, p = 0.071. Additionally, PLWH and hemorrhagic stroke were more likely to be females, 66.7% (10/16) vs 37.3% (41/110) in PWOH, p = 0.031, Table 3.

### Stroke outcomes

#### In-hospital outcomes and associated complications

The overall in-hospital mortality was 31.8% (94/295), and almost half developed sepsis on the ward, 49.2% (145/295). Sepsis was more common among PWOH 51.3% (138/269) vs 26.9% (7/26) in PLWH, p = 0.015. PLWH were more likely to have a longer hospital stay, 5 days (IQR 2–7) vs 4 days (IQR 3–7), compared to PWOH, p = 0.001, Table 4.

#### 6-month outcomes

**Fig. 2.**
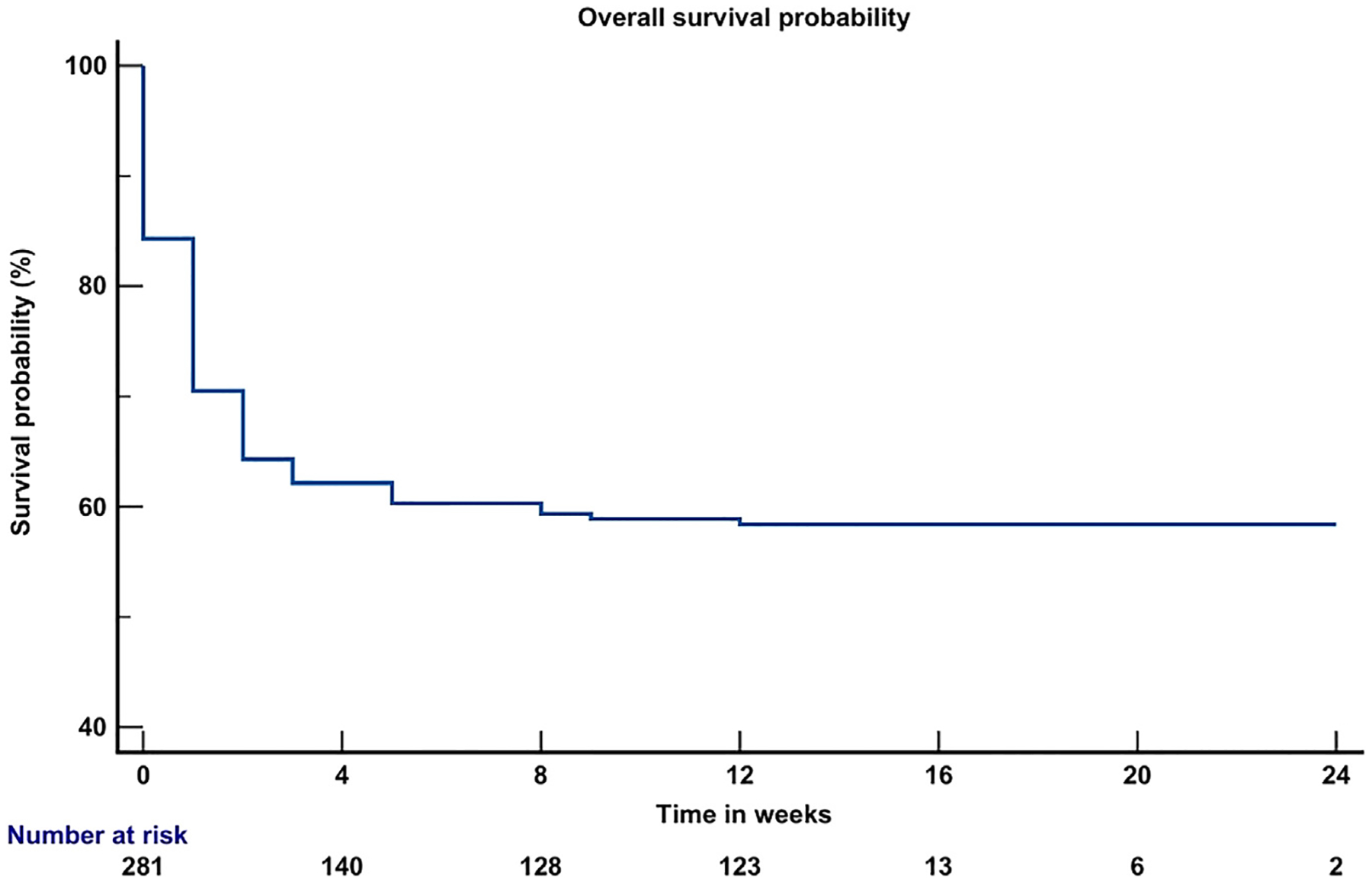
Kaplan Meier overall survival probability.

We found unfavorable 6-month functional outcomes (mRS 3–6) in both groups, 84.7% (250/295). There was no statistically significant difference in the mortality between PLWH and POWH, 42.3% (11/26) vs 38.3% (103/269), p = 0.128, Table 4. In Kaplan-Meier analysis, a total of 95.3% (281/295) of adults were eligible for survival analysis, and the remaining 4.7% (14/295) were missing time to event. The overall mortality rate at 6 months was 38.6% (95% CI 32.6%, 43.8%). The median survival was 14.8 weeks (95% CI 13.4–16.1), as shown in Fig. 2.

#### Predictors of mortality

**Fig. 3.**
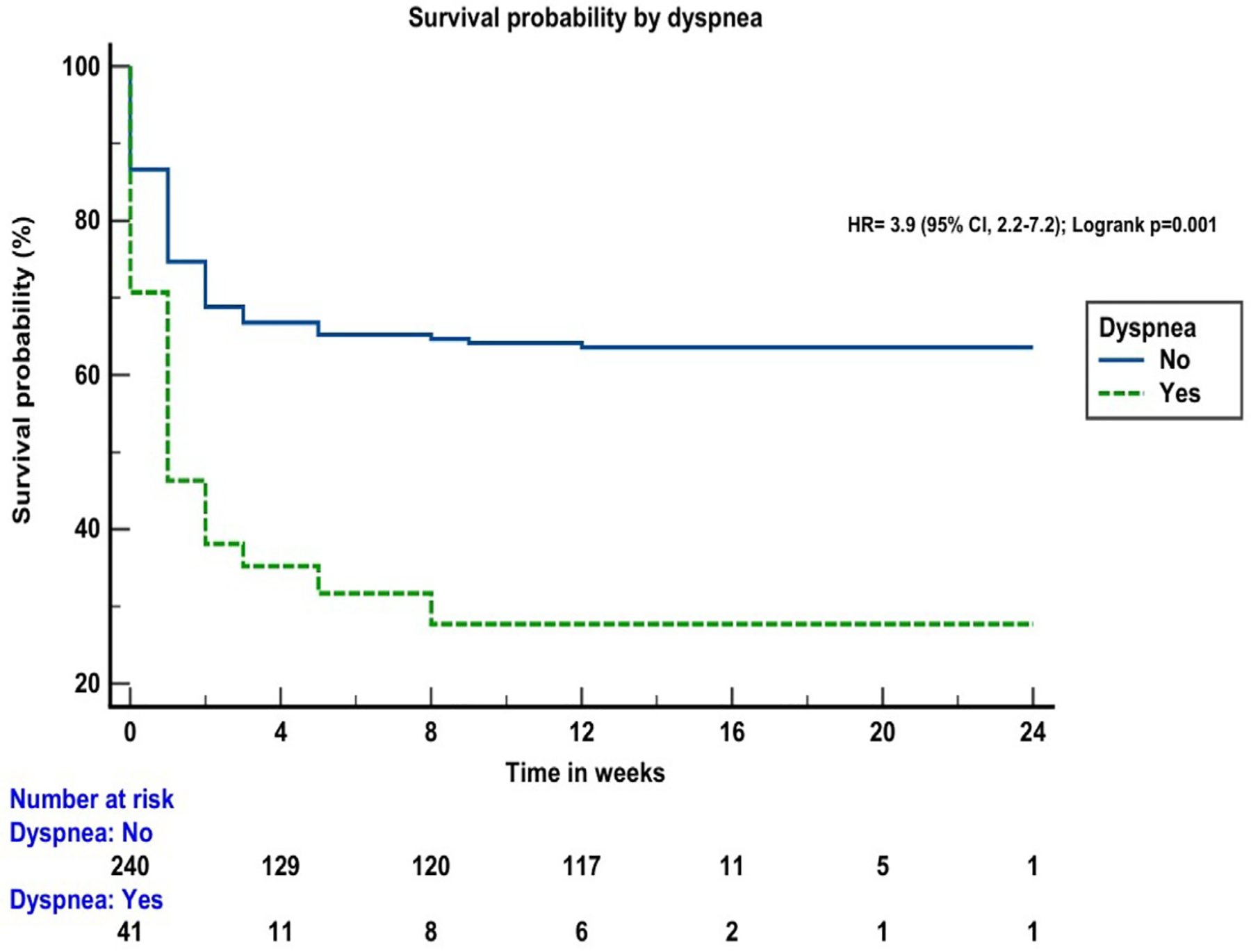
Kaplan Meier survival probability by dyspnea.

**Fig. 4.**
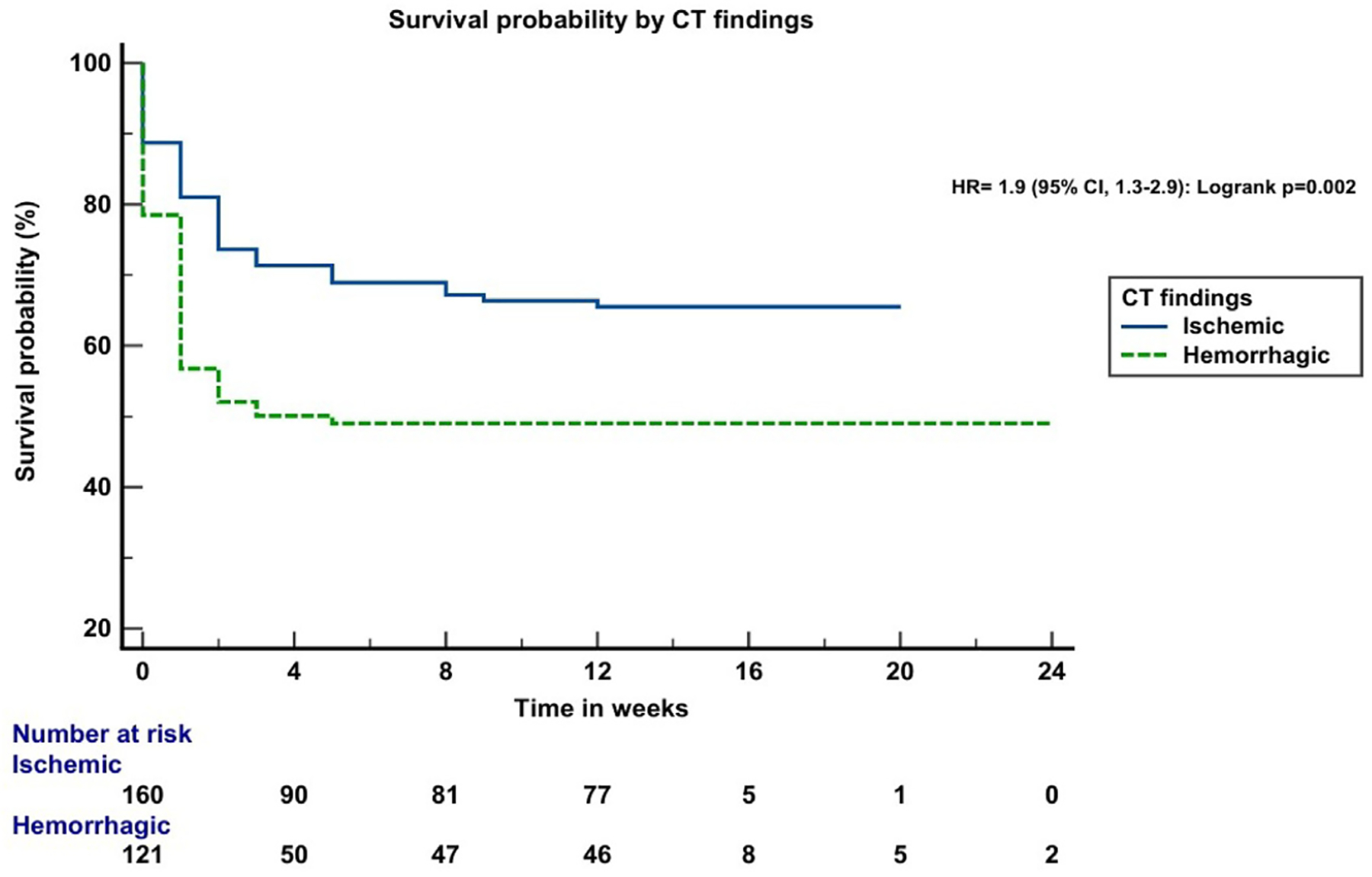
Kaplan Meier survival probability by CT findings.

**Fig. 5.**
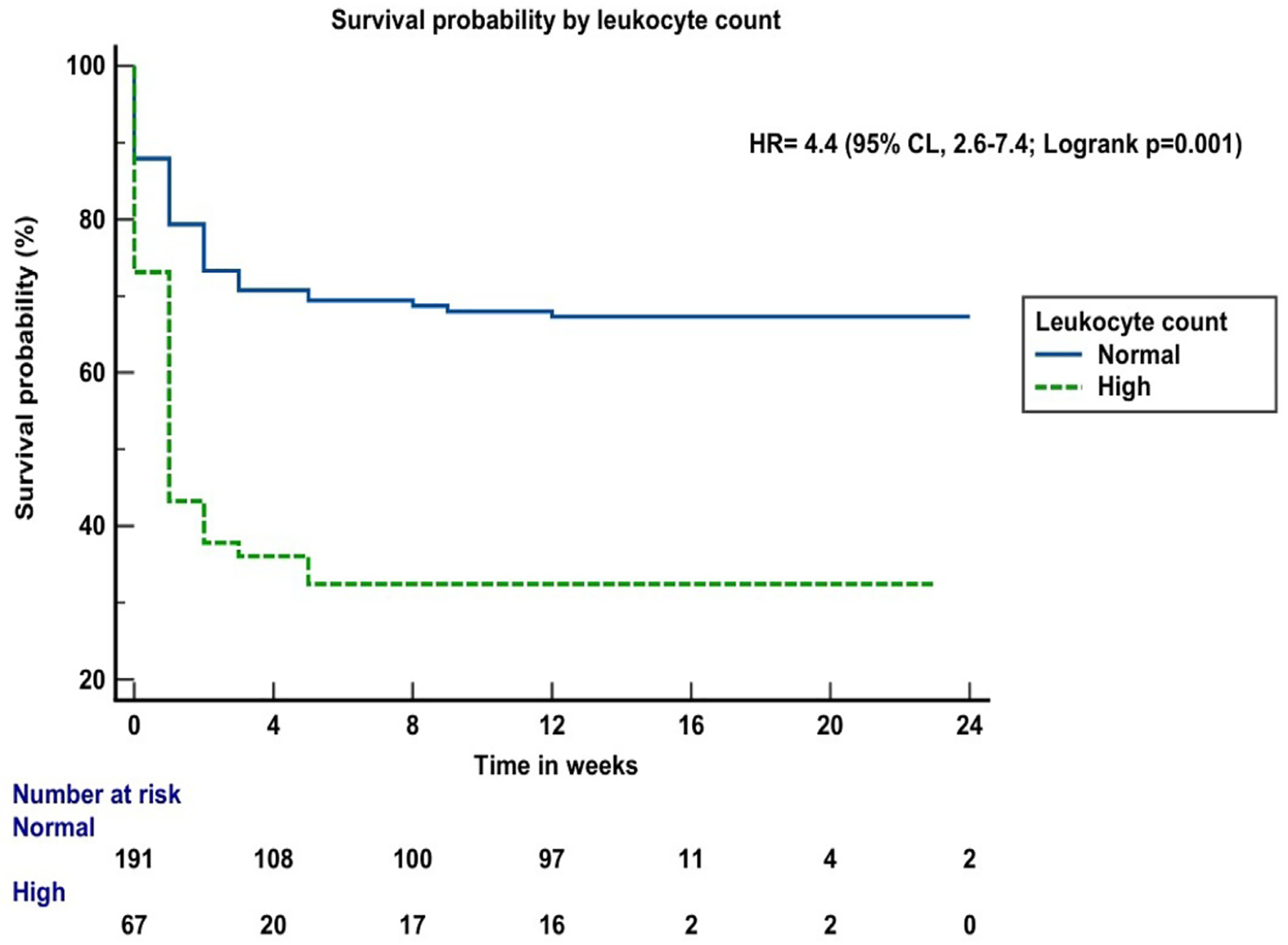
Kaplan Meier survival probability by leukocyte count.

**Fig. 6.**
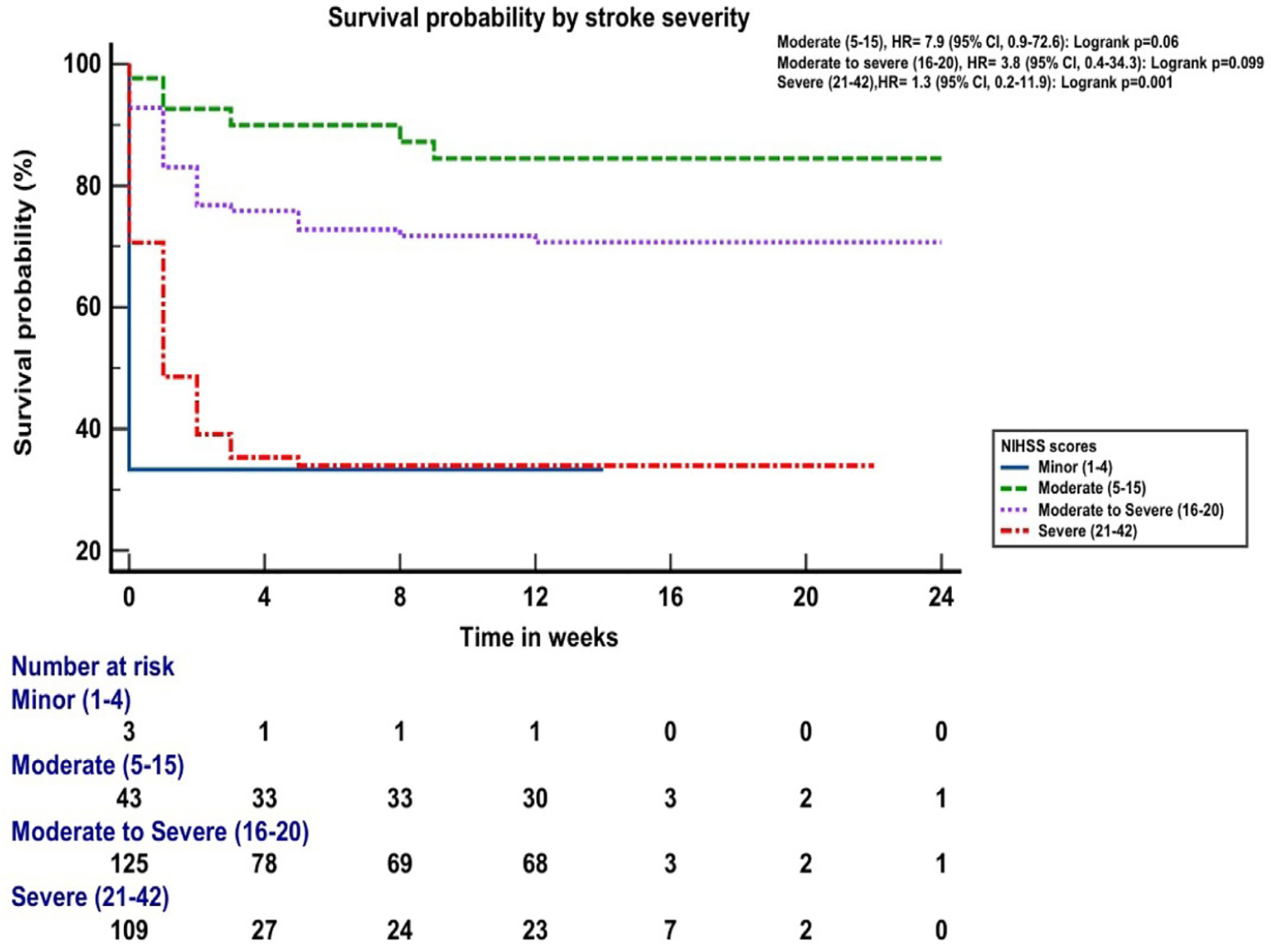
Kaplan Meier survival probability by stroke severity based on NIHSS scores.

In the multivariable Cox regression hazard model, 273 of the 295 participants (92.5%) had complete data and were included in the final analysis. Given the low proportion of missing data (<7.5%), a complete-case analysis was performed and no imputation was undertaken. Dyspnea at presentation (HR 1.6, 95%CI 1.1–2.7, p = 0.046), presence of leukocytosis on admission (aHR 2.2, 95%CI 1.4–3.4, p = 0.003), and severe stroke (NIHSS scores 21–42) (aHR 2.2, 95%CI 1.4–3.7, p = 0.001), were independent predictors of 6-month mortality. While HIV status (aHR 0.8, 95%CI 0.4–1.7, p = 0.602) was not an independent predictor of 6-month mortality, Table 5. No evidence of significant multicollinearity was identified, with all tolerance values > 0.7 and all VIF values < 1.3. Fig. 3,4,5,6

## Discussion

In the present study, we found an HIV proportion of 8.8% among adult patients admitted with stroke at a tertiary teaching hospital in Northwestern Tanzania. Our key findings indicate that stroke in HIV patients was more likely to occur at a younger age, in females, and was associated with hypertension. Moreover, our findings showed PLWH had similar stroke severity, 6-month functional outcomes, and mortality trends to those without HIV.

Our findings showed a HIV proportion of 8.8% among adult patients admitted with stroke, twice the current general population HIV prevalence of 4.4% in Tanzania reported by the Tanzanian HIV Impact Survey (THIS 2022–2023). These findings, therefore, still emphasize the need for reinforced prevention and early detection strategies tailored to PLWH. Nevertheless, our findings indicate a significant drop in the proportion of HIV associated stroke compared to that seen in a previous study done 15 years ago in Tanzania, which reported a prevalence of 20.4% [16]. This is explained by the effectiveness of public health initiatives on HIV prevention, which have resulted in a decline of over 47% of new HIV infections in Tanzania since 2010 [39]. Besides, the widespread, effective use of ARTs has revolutionized the treatment landscape for HIV, improved the quality of life, and reduced AIDS associated complications [5]. These findings closely resemble previous studies done in Sierra Leone (2019–2021) and Tygerberg Hospital in South Africa (2019) that showed a proportion of 7.1% and 9.3% among all patients admitted with stroke, respectively [40,11].

Our data showed that stroke occurs at a younger age in PLWH, 53.5 years (IQR 44.3–67.0) vs 66.0 years (IQR 54.0–78.0) in PWOH, p = 0.001. A significantly younger age compared to PWOH may influence differences in clinical presentation and outcomes. Our findings may be attributed to the added burden of immunological and traditional risk factors in PLWH at an age where strokes are still rare in the general population [41]. Moreover, HIV infection itself may contribute to the development of stroke via several mechanisms, including chronic inflammation, immune system dysfunction, and potential adverse effects of ARTs [42]. A retrospective study done in the US reported the median age of stroke in HIV to be 43 years in 1997 and 48 years in 2006 [43]. In a recent study done in Uganda, age distribution among people living with HIV was younger, with the mean age of 49 vs 59 years among people without HIV [15]. Stroke in younger HIV patients may lead to long-term neurological deficits impacting quality of life, productivity, and psychosocial well-being [44]. A focused and comprehensive stroke prevention in PLWH is necessary. This age disparity may also contribute to the broader variability in age observed across the cohort, reflecting heterogeneity in stroke presentation between PLWH and PWOH.

This study reports stroke in PLWH occurred more often in females (61.5%) than males (38.5%), which is similar to a study done looking at sex differences in the risk of stroke associated with traditional and non-traditional factors in PLWH, and concluded that female sex was associated with higher risk of stroke HR 2.01 (95% CI, 1.25–3.21) at age 40 vs HR 0.60 (95% CI, 0.34–1.06) at age 60 [45]. The sex disparity may be attributed to various biological and socio-cultural elements. Biologically, HIV infection results in chronically elevated immune activation and endothelial dysfunction that accelerate earlier menopause, compounding stroke risk [46]. Social-culturally, women living with HIV often face barriers in healthcare engagement, adherence, and screening for cardiovascular risk factors due to stigma, caregiving responsibilities, or limited economic resources [47]. Therefore, there is a need for an inclusive approach to addressing stroke risk factors in people living with HIV based on the unique challenges faced by female patients.

Stroke in PLWH has been increasingly linked to traditional risk factors, which aligns with our study findings [40,48,49]. In this study, hypertension was the most common traditional risk factor (57.6%) among PLWH with stroke, exceeding diabetes, smoking, alcohol consumption, previous stroke, and cardiovascular diseases. Similarly, a review on the characteristics, prevention, and management of cardiovascular diseases (CVDs) in PLWH highlighted that traditional risk factors contribute to cardiovascular diseases, including stroke, indicating the relationship between HIV infection and non-communicable diseases (NCDs) [50,51]. In addition, despite well-controlled HIV, the median CD4 count of 518 cells/μl (IQR 151.5–571), our patients still carried a double burden of NCDs, commonly hypertension. Hypertension management should be integrated into routine HIV care to prevent stroke and associated CVDs.

Hemorrhagic stroke was the most common neuroimaging finding (57.7%) among PLWH, with ICH being the predominant hematoma location (38.5%); however, this difference was not statistically significant. This is similar to previous studies, which revealed a proportion of ICH located in the subcortical regions, indicating hypertensive vasculopathy [52]. This is further explained by the fact that hypertension is the most common risk factor in our setting [20,48], which is strongly linked with hemorrhagic stroke [53]. On the contrary, most studies suggested ischemic stroke as the most common stroke subtype among people with HIV infection [15,40,48]. This difference in stroke subtypes could stem from the atherosclerotic effects of ART in patients in those studies, whereas most of our patients were newly diagnosed and naïve to ART.

PLWH had lower observed rates of sepsis, 26.9%, compared to PWOH, 51.3%. Given the small number of PLWH in the cohort (n=26), this finding should be interpreted cautiously. The difference may reflect baseline demographic and clinical differences between groups rather than a direct effect of HIV status. PLWH in our cohort were younger and had fewer cardiometabolic comorbidities, factors that independently influence susceptibility to infection risk and post-stroke complications [36,54]. Additionally, over one-third of the initial cohort was excluded due to in-hospital mortality prior to HIV testing or incomplete testing, introducing potential survival and selection bias. We also did not collect detailed data on the timing, source, or microbiological confirmation of sepsis. Taken together, these limitations make it difficult to attribute the observed difference to a biological mechanism. Larger studies with systematic infection characterization are needed to clarify this relationship.

A notable finding in our cohort was a very high proportion of patients presenting with moderate to severe stroke 44.7% and severe stroke 38.6%, with a mean NIHSS score of 19.8 ± 5.8; however, there was no statistically significant difference between PLWH and PWOH. This pattern likely reflects systemic and contextual factors within our setting rather than disease characteristics alone. Delayed hospital presentation was common, with nearly half of patients (48.5%) arriving more than 24 hours after symptom onset, limiting opportunities for early intervention. In addition, BMC is a tertiary referral hospital serving a large catchment population, and many patients are referred from lower-level facilities only after neurological deterioration, introducing a referral bias toward more severe cases. Furthermore, limited public awareness of stroke symptoms, transportation challenges, and financial constraints may further contribute to late arrival and advanced neurological deficits at admission [55,56].

Despite the high proportion of moderate to severe strokes (NIHSS >16) and the high frequency of sepsis in this cohort (49.2%), the median length of hospital stay was relatively short at 4 days (IQR 3–7). This finding should not be interpreted as reflecting rapid neurological recovery. Among patients who developed sepsis, shorter hospital stays may partly reflect early in-hospital mortality rather than clinical improvement. Overall, the observed length of stay likely reflects structural characteristics of stroke care delivery in our setting. Inpatient management primarily emphasizes acute medical stabilization and secondary prevention, while structured multidisciplinary inpatient rehabilitation remains limited. Although physiotherapy services are available, comprehensive rehabilitation, including occupational and speech therapy, is not routinely provided during admission. Consequently, patients are typically discharged once clinically stable and continue recovery at home under family support, with outpatient physiotherapy when accessible.

Additionally, 60.7% of patients in this cohort lacked health insurance coverage, which may influence access to inpatient services, essential medications, and post-discharge rehabilitation resources. This interpretation is supported by recent regional data demonstrating that lack of health insurance is independently associated with higher post-stroke fatality and that engagement with follow-up care and physiotherapy remains low in this setting [12]. Together, these contextual health system factors help explain the relatively short hospital stay despite substantial neurological deficits, high complication rates, and elevated mortality.

Our findings indicated that both PLWH and PWOH groups experienced unfavourable 6-month outcomes (mRS 3–6), 84.7% (250/295), and a high 6-month mortality rate of 38.6% (95% CI (32.6%, 43.8%). We acknowledge that dichotomizing mRS into favourable (0–2) and unfavourable (3–6) outcomes may mask important differences within the unfavourable outcome group, particularly given the high proportion of disability observed at 6 months in this cohort. This is consistent with our recent research findings that suggested stroke mortality of 43.0% (95% CI, 36.6%, 49.4%) at 1 year [12].

In PLWH not on ART, hospitalized with opportunistic infections, unfavorable outcomes, and higher in-hospital mortality were reported [57]. In the post-ART era however, Mbonde et al reported that PLWH presenting with stroke had significantly less disability and lower odds of 90-day mortality than their counterparts without HIV, demonstrating the protective effect of ART and routine care and treatment clinic (CTC) visits [15]. Predictors of overall 6-month survival were more likely to be due to dyspnea, leukocytosis, and severe stroke at admission, parallel with several hospital-based studies done among adults with stroke [58–60]. Moreover, HIV status was not found to be an independent predictor of 6-month mortality in the multivariable analysis despite the high proportion of newly diagnosed and ART-naïve patients in the cohort. This finding should be interpreted in the context of strong competing predictors of mortality identified in our model, including stroke severity, dyspnea at presentation, and leukocytosis, as well as potential survival and selection bias related to the exclusion of patients who died before HIV testing or had incomplete testing. These findings suggest that acute clinical severity and systemic complications, rather than HIV status alone, are the primary drivers of short-term mortality following stroke in this setting. Accordingly, comprehensive clinical assessment, including stroke severity, respiratory evaluation, and routine laboratory testing, remains essential at admission.

This study had several limitations. It was a single-center and hospital-based study, therefore limiting the generalizability of our findings. A substantial proportion of patients (36.3%) were not tested for HIV due to in-hospital mortality prior to testing, stroke mimics, or incomplete HIV testing. This may have introduced survival and selection bias, potentially leading to underestimation of the true proportion of HIV in the cohort and bias in comparative analyses of mortality and complications, including sepsis, between PLWH and PWOH.

CD4+ cell counts were unavailable for many patients because of high in-hospital mortality. We did not evaluate the use of antiretroviral therapies, including regimen and duration, as the largest percentage of our cohort were newly diagnosed and had not yet initiated ART. An incomplete etiologic evaluation limited the application of the TOAST classification to all ischemic stroke cases. Etiologic subtyping was based on routine clinical investigations available within standard care, and advanced diagnostic tests required to reliably identify less common causes (e.g., immune-mediated, infectious, substance-related etiologies, or prolonged cardiac rhythm abnormalities) were not routinely available. Consequently, certain rare causes of ischemic stroke may have been under-ascertained, particularly within the category of other determined etiology. Furthermore, vessel imaging, including CT angiography, carotid Doppler ultrasound, and TTE were performed only when clinically indicated rather than a routine screening (Table S2). As a result, some cases of underlying intracranial and extracranial atherosclerotic disease, cardioembolic sources, or other vascular abnormalities may not have been identified. This may have resulted in misclassification of stroke etiology and affected the distribution of TOAST subtypes reported in the study. Additionally, the missing data in the functional outcomes (mRS), residency, health insurance, and admission NIHSS variables were primarily due to incomplete documentation.

We did not collect detailed data on the source, timing, or microbiological confirmation, which limits the interpretation of the observed differences in sepsis rates between groups. Our comparison between included and excluded patients demonstrated that excluded patients differed in several baseline characteristics, including age, residency, education level, previous stroke, and premorbid functional status (all p < 0.05). These findings indicate that exclusion was not random and primarily reflected differences in baseline vulnerability and vascular risk burden rather than acute stroke severity, as admission NIHSS categories and in-hospital mortality did not differ significantly between groups. Consequently, this selection pattern may have influenced prevalence estimates and comparative analyses by HIV status and should be considered when interpreting study findings.

## Conclusion

In our stroke registry, 9% of registered stroke cases occurred in people living with HIV. However, stroke characteristics among PLWH showed similar severe stroke at presentation, unfavorable outcomes, and 6-month mortality trends to PWOH, with a trend toward more hemorrhagic stroke in PLWH. Our findings suggest that more work is needed to both prevent stroke and improve stroke outcomes in PLWH. Future research should continue to explore the underlying mechanisms and develop targeted strategies to optimize stroke outcomes in this population.

## Supplementary Material

Supplementary Information

Supplementary material associated with this article can be found, in the online version, at https://doi.org/10.1016/j.neuros.2026.100063.

## Figures and Tables

**Table 1:** Comparison of baseline characteristics among PLWH and PWOH.

Variables	Total N=295	PLWH N=26 (%)	PWOH N=269 (%)	p-value
Age (years)				
Median (IQR)	66.0 (53.0–77.0)	53.5 (44.3–67.0)	66.0 (54.0–78.0)	0.001
Gender				
Male	165	10 (38.5)	155 (57.6)	0.066
Female	130	16 (61.5)	114 (42.8)	
Health Insurance, n=294				
Yes	115	8 (30.8)	107 (39.8)	0.354
No	179	18 (69.2)	161 (60.0)	
Referral status				
Referred	206	18 (69.2)	188 (69.9)	0.388
Self-referred	89	8 (30.8)	81 (30.1)	
Marital status				
Married	291	26 (100.0)	265 (98.5)	0.389
Not married	4	0 (0.0)	4 (1.5)	
Residency, n=253				
Urban	90	8 (30.8)	82 (30.9)	0.502
Semi-urban	114	11 (42.3)	103 (38.3)	
Rural	49	2 (7.7)	47 (17.5)	
Level of education, n=295				
None	61	0 (0.0)	61 (22.7)	0.002
Primary	121	13 (50.0)	108 (40.1)	
Secondary	78	11 (42.3)	67 (24.9)	
College and above	35	2 (7.7)	33 (12.6)	
Onset to Door Time (OTD)				
<1 hour	2	0 (0.0)	2 (0.7)	0.634
1–4.5 hours	12	2 (7.7)	10 (3.7)	
4.5–6 hours	10	0 (0.0)	10 (3.7)	
6–12 hours	34	4 (15.4)	30 (11.2)	
12–24 hours	90	7 (26.9)	83 (30.6)	
>24 hours	143	13 (50.0)	130 (48.3)	
Stroke risk factor				
Hypertension	205	15 (57.6)	190 (70.6)	0.183
On regular treatment	80	5 (19.2)	75 (27.9)	0.584
Diabetes Mellitus	42	1 (3.8)	41 (15.2)	0.068
On regular treatment	27	0 (0.0)	27 (10.0)	0.363
Smoking	1	0 (0.0)	1 (0.4)	0.667
Alcohol intake	4	0 (0.0)	4 (1.5)	0.389
Previous stroke	27	2 (7.7)	25 (9.3)	0.782
Cardiac diseases	9	0 (0.0)	9 (3.3)	0.26
CT findings				
Ischemic	147	8 (30.8)	139 (51.7)	0.283
Hemorrhagic	125	15 (57.7)	110 (40.9)	
Normal	18	2 (7.7)	16 (5.9)	
Ischemic with hemorrhagic transformation	5	1 (3.8)	4 (1.5)	
TOAST -classifiable cases n=90				
Large vessel atherosclerosis	71	3 (11.5)	68 (25.3)	0.187
Cardio embolism	6	0 (0.0)	6 (2.2)	
Small vessel occlusion	12	2 (7.7)	10 (3.7)	
Other determined etiology	0	0 (0.0)	0 (0.0)	
Undetermined etiology	1	0 (0.0)	1 (0.4)	
Not classifiable by TOAST				
Unclassified due to insufficient etiologic data, n=62	62	4 (15.4)	58 (21.6)	
Hemorrhagic location, n=125				
ICH	94	10 (38.5)	84 (31.2)	0.826
SH	15	3 (11.5)	12 (4.5)	0.137
IVH	16	2 (7.7)	14 (5.2)	0.641
Pre-morbid mRS, n=295				
No symptoms (score 0)	221	15 (57.6)	206 (76.6)	0.006
No significant disability (score 1)	9	0 (0.0)	9 (3.3)	
Slight disability (score 2)	14	2 (7.7)	12 (4.5)	
Moderate disability (score 3)	24	4 (15.4)	20 (7.1)	
Moderate to severe disability (score 4)	18	2 (7.7)	16 (5.9)	
Severe disability (score 5)	9	3 (11.5)	6 (2.2)	
Admission stroke severity-NIHSS, n=295				
Minor (1–4)	4	1 (3.8)	3 (1.1)	0.478
Moderate (5–15)	45	2 (7.7)	43 (15.9)	
Moderate to severe (16–20)	132	12 (46.2)	120 (44.6)	
Severe (21–42)	114	11 (42.3)	103 (38.3)	
Admission stroke severity-NIHSS				
Mean (SD)	19.8 ± 5.8	19.9 ± 5.3	19.8 ± 5.9	0.552
Arrival RBG (mmol/l)				
Mean (SD)	7.8 ± 3.4	8.0 ± 4.3	7.8 ± 3.3	0.704
Leukocyte counts (10^9^ /L)				
Median (IQR)	8.7 (6.8–10.9)	6.9 (5.8–12.3)	8.7 (6.8–11.0)	0.298
Low-Density Lipoprotein (mg/dL)				
Median (IQR)	131 (104–162)	101 (58–143)	131 (104–162)	0.037
CD4 counts (cells/μl)				
Median (IQR)	—	518 (151.5–571)	—	—

PLWH- People living with HIV, PWOH- People without HIV, mRS- modified Rankin Scale, NIHSS- National Institute of Health Stroke Scale, ICH- Intracerebral Hemorrhage, SH- Subarachnoid Hemorrhage, IVH- Intraventricular Hemorrhage, OTD- Onset to Door Time, IQR- Interquartile Range, N- number, IQR- Interquartile range, RBG- Random Blood Glucose, TOAST-Trial of Org 10172 in Acute Stroke Treatment classification. TOAST classification was applied only to patients with confirmed ischemic stroke (including the 5 with hemorrhagic transformation, as the underlying etiology is ischemic). Of the 152 ischemic strokes, 90 had completed a predefined minimum etiologic evaluation for TOAST classification. Sixty-two participants lacked sufficient etiologic data and were therefore not classified according to TOAST criteria.

**Table 2:** Baseline characteristics of participants with confirmed imaging findings of ischemic stroke, stratified by HIV status.

Variables	Total N=147	PLWH N=8 (%)	PWOH N=139 (%)	p-value
Age (years)				
Median (IQR)	70.0 (60.0–79.0)	59.0 (49.5–70.0)	70.0 (62.0–80.0)	0.016
Gender				
Male	77	4 (50.0)	73 (52.5)	0.890
Female	70	4 (50.0)	66 (47.5)	
Stroke risk factor				
Hypertension	105	5 (62.5)	100 (71.9)	0.205
On regular treatment	48	1 (12.5)	47 (33.8)	0.374
Diabetes Mellitus	28	1 (12.5)	27 (19.4)	0.611
On regular treatment	16	0 (0.0)	16 (11.5)	0.199
Smoking	0	0 (0.0)	0 (0.0)	—
Alcohol intake	1	0 (0.0)	1 (0.7)	0.738
Previous stroke	22	1 (12.5)	21 (15.1)	0.111
Cardiac diseases	7	0 (0.0)	7 (5.0)	0.304

PLWH- People living with HIV, PWOH- people without HIV, N-number, IQR- Interquartile range.

**Table 3:** Baseline characteristics of participants with confirmed imaging findings of hemorrhagic stroke, stratified by HIV status.

Variables	Total N=125	PLWH N=15 (%)	PWOH N=110 (%)	p-value
Age (years)				
Median (IQR)	58.0 (48.0–68.5)	49.0 (42.0–64.0)	59.0 (48.0–69.9)	0.071
Gender				
Male	74	5 (33.3)	69 (62.7)	0.031
Female	51	10 (66.7)	41 (37.3)	
Stroke risk factor				
Hypertension	87	10 (66.7)	77 (70.0)	0.183
On regular treatment	32	4 (26.7)	28 (25.5)	0.756
Diabetes Mellitus	12	0 (0.0)	12 (10.9)	0.072
On regular treatment	9	0 (0.0)	9 (8.2)	0.316
Smoking	1	0 (0.0)	1 (0.9)	0.612
Alcohol intake	2	0 (0.0)	2 (1.8)	0.473
Previous stroke	5	1 (6.7)	4 (3.6)	0.602
Cardiac diseases	2	0 (0.0)	2 (1.8)	0.612

PLWH- People living with HIV, PWOH- people without HIV, N-number, IQR- Interquartile range.

**Table 4:** In-hospital and 6-month complications and functional outcomes.

Variables	Total N=295	PLWH N=26 (%)	PWOH N=269 (%)	p-value
Length of hospital stay				
Median (IQR) in days	4 (3–7)	5 (2–7)	4 (3–7)	0.001
In-hospital complications				
Aspiration pneumonia	81	5 (19.2)	76 (28.3)	0.304
Deep venous thrombosis	4	1 (3.8)	3 (1.1)	0.336
Urinary tract infection	45	3 (11.5)	42 (15.6)	0.563
Pulmonary embolism	12	1 (3.8)	11 (4.1)	0.949
Sepsis	145	7 (26.9)	138 (51.3)	0.015
Kidney injury	86	8 (30.8)	78 (28.9)	0.859
Hyponatremia	54	3 (11.5)	51 (18.9)	0.321
Other complications	10	2 (7.7)	8 (3.1)	0.267
No complications	66	8 (30.8)	58 (21.6)	0.303
Discharge mRS, n=287				
No symptoms (score 0)	4	0 (0.0)	4 (1.5)	0.128
No significant disability (score 1)	2	1 (3.8)	1 (0.4)	
Slight disability (score 2)	25	0 (0.0)	25 (9.3)	
Moderate disability (score 3)	100	11 (42.3)	89 (33.1)	
Moderate to severe disability (score 4)	57	3 (11.5)	54 (20.1)	
Severe disability (score 5)	5	1 (3.8)	4 (1.5)	
Dead (score 6)	94	10 (38.5)	84 (31.2)	
6-month mRS, n=287				
No symptoms (score 0)	4	1 (3.8)	3 (1.1)	0.248
No significant disability (score 1)	6	1 (3.8)	5 (1.9)	
Slight disability (score 2)	27	0 (0.0)	27 (10.0)	
Moderate disability (score 3)	83	8 (30.8)	75 (27.9)	
Moderate to severe disability (score 4)	50	4 (15.4)	46 (17.1)	
Severe disability (score 5)	3	1 (3.8)	2 (0.7)	
Dead (score 6)	114	11 (42.3)	103 (38.3)	
6-month outcome				
(score 0–2)	37	2 (7.7)	35 (13.0)	0.549
(score 3–6)	250	24 (92.3)	226 (87.0)	

mRS- modified Rankin Scale, PLWH- people living with HIV, PWOH- people without HIV, N-number, SD- standard deviation, IQR- Interquartile Range.

**Table 5:** Cox proportional hazard for predictors of overall 6-month mortality.

Variable	HR (95% CI)	p-value	aHR (95% CI)	p-value
Age				
18–64 years	REF			
> 65 years	1.2 (0.8–1.8)	0.296	1.3 (0.8–2.1)	0.234
Gender				
Males	REF			
Females	0.9 (0.6–1.3)	0.628	0.8 (0.5–1.2)	0.281
Health insurance				
Yes	REF			
No	1.2 (0.8–1.8)	0.355	0.9 (0.6–1.4)	0.743
Referral status				
Not referred	REF			
Referred	1.1 (0.7–1.6)	0.808	0.7 (0.4–1.1)	0.103
HIV status				
Negative	REF			
Positive	1.0 (0.5–1.9)	0.952	0.8 (0.4–1.7)	0.602
Dyspnea				
Absent	REF			
Present	3.9 (2.2–7.2)	0.001	1.6 (1.1–2.7)	0.046
CT findings				
Ischemic	REF			
Hemorrhagic	1.9 (1.3–2.9)	0.002	1.5 (0.9–2.4)	0.081
Admission NIHSS				
Minor (1–4)	REF			
Moderate (5–15)	7.9 (0.9–72.6)	0.06	4.3 (0.9–18.6)	0.05
Moderate to severe (16–20)	3.8 (0.4–34.3)	0.099	0.5 (0.2–1.1)	0.089
Severe (21–42)	1.3 (0.2–11.9)	0.001	2.2 (1.4–3.7)	0.001
Leukocyte Count				
(4.5–11.0) × 10^9^ /L	REF			
> 11.1×10^9^ /L	4.4 (2.6–7.4)	0.001	2.2 (1.4–3.4)	0.003

NIHSS- National Institute of Health Stroke Scale, HIV- Human Immunodeficiency Virus.

## List of abbreviations

DALYs: Disability Adjusted Life Years
AIDS: Acquired immunodeficiency syndrome
HIV: Human immunodeficiency Virus
ART: Antiretroviral therapy
PLWH: People Living with HIV
PWOH: People without HIV
CTC: Care and Treatment Clinic
mRS: modified Rankin Scale
NIHSS: National Institutes of Health Stroke Scale

## References

[1] Feigin VL, Brainin M, Norrving B, Martins S, Pandian JD, Lindsay P, Fredin Grupper MM, Rautalin I. World Stroke Organization (WSO): Global Stroke Fact Sheet 2025. Int J Stroke 2025;0(ja):17474930241308142. doi:10.1177/17474930241308142.

[2] Feigin VL, Abate MD, Abate YH, Abd ElHafeez S, Abd-Allah F, Abdelalim A, Abdelkader A, Abdelmasseh M, Abd-Elsalam S, Abdi P, Abdollahi A, Abdoun M, Abd-Rabu R, Abdulah DM, Abdullahi A, Abebe M, Abeldaño Zuñiga RA, Abhilash ES, Abiodun OO, Murray CJL. Global, regional, and national burden of stroke and its risk factors, 1990&2013;2021: a systematic analysis for the Global Burden of Disease Study 2021. Lancet Neurol 2024;23(10):973–1003. doi:10.1016/S1474-4422(24)00369-7. 39304265 PMC12254192

[3] Mudie K, Jin MM, Tan, Kendall L, Addo J, Dos-Santos-Silva I, Quint J, Smeeth L, Cook S, Nitsch D, Natamba B, Gomez-Olive FX, Ako A, Perel P. Non-communicable diseases in sub-Saharan Africa: a scoping review of large cohort studies. J Glob Health 2019;9(2):020409. doi:10.7189/jogh.09.020409. 31448113 PMC6684871

[4] Prust ML, Forman R, Ovbiagele B. Addressing disparities in the global epidemiology of stroke. Nat Rev Neurol 2024;20(4):207–21. doi:10.1038/s41582-023-00921-z. 38228908

[5] Saag MS, Gandhi RT, Hoy JF, Landovitz RJ, Thompson MA, Sax PE, Smith DM, Benson CA, Buchbinder SP, Del Rio C, Eron JJ Jr, Fätkenheuer G, Günthard HF, Molina JM, Jacobsen DM, Volberding PA. Antiretroviral drugs for treatment and prevention of HIV infection in adults: 2020 recommendations of the International Antiviral Society-USA Panel. Jama 2020;324(16):1651–69. doi:10.1001/jama.2020.17025. 33052386 PMC11017368

[6] Walker R, Whiting D, Unwin N, Mugusi F, Swai M, Aris E, Jusabani A, Kabadi G, Gray WK, Lewanga M, Alberti G. Stroke incidence in rural and urban Tanzania: a prospective, community-based study. Lancet Neurol 2010;9(8):786–92. doi:10.1016/s1474-4422(10)70144-7. 20609629

[7] Benjamin LA, Bryer A, Emsley HC, Khoo S, Solomon T, Connor MD. HIV infection and stroke: current perspectives and future directions. Lancet Neurol 2012;11(10):878–90. doi:10.1016/s1474-4422(12)70205-3. 22995692 PMC3460367

[8] Cole JW, Pinto AN, Hebel JR, Buchholz DW, Earley CJ, Johnson CJ, Macko RF, Price TR, Sloan MA, Stern BJ, Wityk RJ, Wozniak MA, Kittner SJ. Acquired immunodeficiency syndrome and the risk of stroke. Stroke 2004;35(1):51–6. doi:10.1161/01.Str.0000105393.57853.11. 14684782

[9] Rabinstein AA. Stroke in HIV-infected patients: a clinical perspective. Cerebrovasc Dis 2003;15(1–2):37–44. doi:10.1159/000067120. 12499709

[10] Singer EJ, Valdes-Sueiras M, Commins DL, Yong W, Carlson M. HIV stroke risk: evidence and implications. Ther Adv Chronic Dis 2013;4(2):61–70. doi:10.1177/2040622312471840. 23556125 PMC3610259

[11] Corbett C, Brey N, Pitcher RD, O’Hagan S, Esterhuizen TM, Chow FC, Decloedt EH. Prevalence and characteristics of HIV-associated stroke in a tertiary hospital setting in South Africa. Neurology 2022;99(9):e904–15. doi:10.1212/WNL.0000000000200780. 36038281 PMC9502736

[12] Three-year post-stroke outcomes in urban North-western Tanzania Ngimbwa J, Ngimbwa J, Nchasi G, Paul IK, Kasala A, Mwamba LA, Berling S, Basinda MK, Xavier G, Andrew B, Mawazo A, Lucas D, Mahawish K, Rudovick L, Wajanga B, Peck R, Matuja SS. (2025). Three-year post-stroke outcomes in urban North-western Tanzania. Front Stroke 2025;4:1593092. doi:10.3389/fstro.2025.1593092. 41541864 PMC12802649

[13] Bearden DR, Omech B, Rulaganyang I, Sesay SO, Kolson DL, Kasner SE, Mullen MT. Stroke and HIV in Botswana: A prospective study of risk factors and outcomes. J Neurol Sci 2020;413:116806. doi:10.1016/j.jns.2020.116806. 32244092

[14] Heikinheimo T, Chimbayo D, Kumwenda JJ, Kampondeni S, Allain TJ. Stroke outcomes in Malawi, a country with high prevalence of HIV: a prospective follow-up study. PLoS One 2012;7(3):e33765. doi:10.1371/journal.pone.0033765. 22479439 PMC3315584

[15] Mbonde AA, Chang J, Musubire AK, Okello S, Kayanja A, Moses A, Butterfield RJ, Chow FC, Saylor DR, O’Carroll CB, Siedner M. HIV infection and 90-day stroke outcomes in Uganda: A prospective observational cohort study. Neurol Clin Pr 2023;13(6):e200198. doi:10.1212/CPJ.0000000000200198. PMC10942001 38495078

[16] Mlay (2010). The prevalence of HIV among patients admitted with stroke at the Muhimbili National Hospital, Dar es Salaam, Tanzania.

[17] Matuja SS, Ngimbwa J, Andrew L, Shindika J, Nchasi G, Kasala A, Paul IK, Ndalahwa M, Mawazo A, Kalokola F, Ngoya P, Rudovick L, Kilonzo S, Wajanga B, Massaga F, Kalluvya SE, Munseri P, Mnacho MA, Okeng’o K, Peck R. Stroke characteristics and outcomes in urban Tanzania: data from the Prospective Lake Zone Stroke Registry. Int J Stroke 2024;19(5):536–46. doi:10.1177/17474930231219584. 38031727 PMC11132936

[18] WHO. (2005). World Health Organization. Noncommunicable Diseases and Mental Health Cluster (2005). WHO STEPS stroke manual : the WHO STEP-wise approach to stroke surveillance /Noncommunicable Diseases and Mental Health, World Health Organization. https://iris.who.int/handle/10665/43420. https://iris.who.int/handle/10665/43420. last accessed 20th November 2025

[19] Yohana C, Bakuza JS, Kinung’hi SM, Nyundo BA, Rambau PF. The trend of schistosomiasis related bladder cancer in the lake zone, Tanzania: a retrospective review over 10 years period. Infect Agent Cancer 2023;18(1):10. doi:10.1186/s13027-023-00491-1. 36800971 PMC9938995

[20] Matuja SS, Nyundo A, Assey E, Bwelemo J, Dekker M, Urasa S, Makarius E, Kishimbo P, Baltazar Y, Alphonce B, Ngimbwa J, Adebayo P, Siha S, Chagula A, Libena M, Lutufyo T, Mnacho MA, Chiwanga FS, Okeng’o K, Matuja W. Early insights from a multi-centre national stroke surveillance initiative in Tanzania. J Stroke Cerebrovasc Dis 2026;35(3):108571. doi:10.1016/j.jstrokecerebrovasdis.2026.108571. 41577313

[21] Alfred H (2014). Population growth, structure and momentum in Tanzania. https://esrf.or.tz/wp-content/uploads/2021/01/THDR-BP-7.pdf

[22] Wenban-Smith HB. Urban Tanzan Popul Growth Intern Migr Urban Tanzan 2014:1967–2012. https://www.theigc.org/sites/default/files/2014/09/Wenban-Smith-2014-Working-Paper.pdf.

[23] Unger T, Borghi C, Charchar F, Khan NA, Poulter NR, Prabhakaran D, Ramirez A, Schlaich M, Stergiou GS, Tomaszewski M, Wainford RD, Williams B, Schutte AE. 2020 International Society of Hypertension Global Hypertension Practice Guidelines. Hypertension 2020;75(6):1334–57. doi:10.1161/HYPERTENSIONAHA.120.15026. 32370572

[24] WHO. (2025). WHO Fact Sheet on hypertension. https://www.who.int/news-room/fact-sheets/detail/hypertension last accessed on February 2026

[25] Committee ADAPP. Diagnosis and classification of diabetes: Standards of Care in Diabetes—2025. Diabetes Care 2024;48(Supplement_1):S27–49. doi:10.2337/dc25-S002. PMC12690183 41358893

[26] Parshall MB, Schwartzstein RM, Adams L, Banzett RB, Manning HL, Bourbeau J, Calverley PM, Gift AG, Harver A, Lareau SC, Mahler DA, Meek PM, O’Donnell DE. An official American Thoracic Society statement: update on the mechanisms, assessment, and management of dyspnea. Am J Respir Crit Care Med 2012;185(4):435–52. doi:10.1164/rccm.201111-2042ST. 22336677 PMC5448624

[27] Suha FM, Sharma P, S. (2025). [Updated 2025 Dec 13]. In: StatPearls [Internet]. Treasure Island (FL): StatPearls Publishing; 2025 Jan-. Available from: https://www.ncbi.nlm.nih.gov/books/NBK499965/.

[28] Berliner D, Schneider N, Welte T, Bauersachs J. The differential diagnosis of dyspnea. Dtsch Arztebl Int 2016;113(49):834–45. doi:10.3238/arztebl.2016.0834. 28098068 PMC5247680

[29] Adams HP Jr, Bendixen BH, Kappelle LJ, Biller J, Love BB, Gordon DL, Marsh EE 3rd. Classification of subtype of acute ischemic stroke. Definitions for use in a multicenter clinical trial. TOAST. Trial of Org 10172 in acute stroke treatment. Stroke 1993;24(1):35–41. doi:10.1161/01.str.24.1.35. 7678184

[30] Crow J, Watt H, Wells M, Malhotra P. Improving follow-up care for people after minor stroke using early personalised care: A protocol for a randomised, mixed-methods, feasibility study. [version 3; peer review: 2 approved]. NIHR Open Res 2025;4(44). doi:10.3310/nihropenres.13649.3. PMC11868739 40027171

[31] Jørgensen JMA, Christensen DL, Nielsen KK, Sadiq HS, Khan MY, Jusabani AM, Walker R. Incidence and characteristics of stroke in Zanzibar-a hospital-based prospective study in a low-income island population. Front Neurol 2022;13:931915. doi:10.3389/fneur.2022.931915. 35968303 PMC9366665

[32] Meyer BC, Hemmen TM, Jackson CM, Lyden PD. Modified National Institutes of Health Stroke Scale for use in stroke clinical trials: prospective reliability and validity. Stroke 2002;33(5):1261–6. doi:10.1161/01.str.0000015625.87603.a7. 11988601

[33] Roth EJ. NIH stroke scale. Encyclopedia of Clinical Neuropsychology. Kreutzer JS, DeLuca J, Caplan B, editors, New York: Springer; 2011. (pp. 1777–1777). doi:10.1007/978-0-387-79948-3_2193.

[34] Martelli (2022). National AIDS Control Programme (NACP). National Comprehensive Guidelines on HIV Testing Services in Tanzania. Vol Third edit. 3d ed. National AIDS Control Program, 2019. https://www.nacp.go.tz/guidelines/

[35] Ganesh A, Luengo-Fernandez R, Wharton RM, Rothwell PM. Oxford sascular study. ordinal vs dichotomous analyses of modified rankin scale, 5-year outcome, and cost of stroke. Neurology 2018;91(21):e1951–60. doi:10.1212/WNL.0000000000006554. 30341155 PMC6260198

[36] Abdallah A, Chang JL, O’Carroll CB, Musubire A, Chow FC, Wilson AL, Siedner MJ. Stroke in Human Immunodeficiency Virus-infected individuals in Sub-Saharan Africa (SSA): a systematic review. J Stroke Cerebrovasc Dis 2018;27(7):1828–36. doi:10.1016/j.jstrokecerebrovasdis.2018.02.016. 29628338 PMC6641537

[37] Getie A, Gedfew M, Kitaw TA, Yilak G, Bimerew M. Mortality rate of stroke and its determinants in Africa: an umbrella review of systematic review and meta-analysis. Glob Epidemiol 2025;10:100225. doi:10.1016/j.gloepi.2025.100225. 41112821 PMC12528930

[38] Moraes MA, Jesus PA, Muniz LS, Baccin CA, Barreto ABM, Sales RS, Pires C, Teles CAS, Mussi FC. Arrival time at a referral hospital and functional disability of people with stroke: a cohort study. Sao Paulo Med J 2023;141(6):e2022510. doi:10.1590/1516-3180.2022.0510.R1.27022023 37194766 PMC10181833

[39] Tanzania-HIV-impact-survey. https://www.cdc.gov/global-health/impact/tanzania-hiv-impact-survey.html. last accessed 12th September 2025

[40] Baldeh M, Youkee D, Lakoh S, Rudd A, Langhorne P, Deen GF, Conteh ZF, Lisk DR, O’Hara J, Thompson M, Brima MT, Wang Y, Wolfe CD, Sackley CM. Stroke in Sierra Leone. The stroke risk factors for people with HIV: A prospective case-control study. J Stroke Cerebrovasc Dis 2023;32(9):107279. doi:10.1016/j.jstrokecerebrovasdis.2023.107279. 37523881 PMC11070751

[41] Allan-Blitz LT, Mena LA, Mayer KH. The ongoing HIV epidemic in American youth: challenges and opportunities. Mhealth 2021;7:33. doi:10.21037/mhealth-20-42. 33898602 PMC8063015

[42] Nou E, Lo J, Grinspoon SK. Inflammation, immune activation, and cardiovascular disease in HIV. Aids 2016;30(10):1495–509. doi:10.1097/qad.0000000000001109. 27058351 PMC4889507

[43] Ovbiagele B, Nath A. Increasing incidence of ischemic stroke in patients with HIV infection. Neurology 2011;76(5):444–50. doi:10.1212/WNL.0b013e31820a0cfc. 21248273 PMC3034413

[44] Patel UK, Malik P, Li Y, Habib A, Shah S, Lunagariya A, Jani V, Dhamoon MS. Stroke and HIV-associated neurological complications: A retrospective nationwide study. J Med Virol 2021;93(8):4915–29. doi:10.1002/jmv.27010. 33837961

[45] Chow FC, Nance RM, Becker K, Ho EL, Huffer A, Kalani R, Marra CM, Zunt JR, Bamford L, Burkholder GA, Cachay E, Eron JJ, Keruly J, Kitahata MM, Napravnik S, Saag MS, Willig AL, Moore RD, Tirschwell DL, Crane HM. Sex differences in the risk of stroke associated with traditional and non-traditional factors in a US cohort of people with HIV infection. Neurology 2024;103(4):e209726. doi:10.1212/wnl.0000000000209726. 39088772 PMC11793864

[46] Lv T, Cao W, Li T. HIV-related immune activation and inflammation: current understanding and strategies. J Immunol Res 2021;2021:7316456. doi:10.1155/2021/7316456. 34631899 PMC8494587

[47] Bouabida K, Chaves BG, Anane E. Challenges and barriers to HIV care engagement and care cascade: viewpoint. Front Reprod Health 2023;5:1201087. doi:10.3389/frph.2023.1201087. 37547803 PMC10398380

[48] Benjamin, HIV, antiretroviral treatment, hypertension, and stroke in Malawian adults: A case-control study. Neurology 2016;86(4):324–33. doi:10.1212/wnl.0000000000002278. 26683649 PMC4776088

[49] Ismael S, Moshahid Khan M, Kumar P, Kodidela S, Mirzahosseini G, Kumar S, Ishrat T. HIV associated risk factors for ischemic stroke and future perspectives. Int J Mol Sci 2020;21(15). doi:10.3390/ijms21155306. PMC7432359 32722629

[50] Grysiewicz RA, Thomas K, Pandey DK. Epidemiology of ischemic and hemorrhagic stroke: incidence, prevalence, mortality, and risk factors. Neurol Clin 2008;26(4):871–95. doi:10.1016/j.ncl.2008.07.003. 19026895

[51] Moawad, Risk of stroke among HIV patients: A systematic review and meta-analysis of global studies and associated comorbidities. J Acquir Immune Defic Syndr 2024;95(5):399–410. doi:10.1097/QAI.0000000000003382. 38489489

[52] Magid-Bernstein J, Girard R, Polster S, Srinath A, Romanos S, Awad IA, Sansing LH. Cerebral hemorrhage: pathophysiology, treatment, and future directions. Circ Res 2022;130(8):1204–29. doi:10.1161/CIRCRESAHA.121.319949. 35420918 PMC10032582

[53] Ariesen MJ, Claus SP, Rinkel GJ, Algra A. Risk factors for intracerebral hemorrhage in the general population: a systematic review. Stroke 2003;34(8):2060–5. doi: 10.1161/01.Str.0000080678.09344.8d. 12843354

[54] Nweke M, Mshunqane N. Characterization and stratification of risk factors of stroke in people living with HIV: A theory-informed systematic review. BMC Cardiovasc Disord 2025;25(1):405. doi:10.1186/s12872-025-04833-2. 40426038 PMC12107966

[55] Gandhi DBC, Mascarenhas R, Zarreen S, Chawla NS, Pandian JD, English C, Solomon JM. Bridging the gap: unique strategies to improve access and implementation of stroke rehabilitation in LMICs - a scoping review. Disabil Rehabil 2025;47(26):6851–63. doi:10.1080/09638288.2025.2495194. 40336256

[56] Martins SCO, Matuja SS. Acute stroke care in low and middle-income countries. Curr Opin Neurol 2025;38(1):47–53. doi:10.1097/wco.0000000000001332. 39508402

[57] Mishore KM, Hussein N, Huluka SA. Hospitalization and predictors of inpatient mortality among HIV-infected patients in Jimma University Specialized Hospital, Jimma, Ethiopia: prospective observational study. AIDS Res Treat 2020;2020:1872358. doi:10.1155/2020/1872358. 32547790 PMC7273427

[58] Kortazar-Zubizarreta I, Pinedo-Brochado A, Azkune-Calle I, Aguirre-Larracoechea U, Gomez-Beldarrain M, Garcia-Monco JC. Predictors of in-hospital mortality after ischemic stroke: A prospective, single-center study. Health Sci Rep 2019;2(4):e110. doi:10.1002/hsr2.110. PMC6482326 31049417

[59] Schwartz L, Anteby R, Klang E, Soffer S. Stroke mortality prediction using machine learning: systematic review. J Neurol Sci 2023;444:120529. doi:10.1016/j.jns.2022.120529. 36580703

[60] Zimba S, Nutakki A, Chishimba L, Chomba M, Bahouth M, Gottesman RF, Saylor D. Risk factors and outcomes of HIV-associated stroke in Zambia. Aids 2021;35(13):2149–55. doi:10.1097/qad.0000000000002999. 34138769

