# Supplementary material for: Stroke in people living with HIV and 6-month outcomes in Northwestern Tanzania
Source: Equity Neurosci. Author manuscript; Available in PMC 2026 Jul 24. (PMC13390899; doi:10.1016/j.neuros.2026.100063)
Supplement: Supplementary Information [file NIHMS2194186-supplement-Supplementary_Information.docx]

**Supplementary Table S1:** Comparison of baseline characteristics among the included and excluded participants

| Variables | Total N=463 | Included participants N=295 (%) | Excluded participants N=168 (%) | p-value |
| --- | --- | --- | --- | --- |
| Age (years) |  |  |  |  |
| Median (IQR) | 64.0 (53.0-75.0) | 65.0 (52.0-76.0) | 63.0 (54.3-74.3) | 0.001 |
| Gender |  |  |  |  |
| Male | 249 | 165 (55.9) | 84 (50.0) | 0.276 |
| Female | 214 | 130 (44.1) | 84 (50.0) |  |
| Health Insurance, n=461 |  |  |  |  |
| Yes | 193 | 115 (39.0) | 78 (46.4) | 0.097 |
| No | 268 | 179 (61.0) | 89 (53.6) |  |
| Residency, n=420 |  |  |  |  |
| Urban | 175 | 90 (30.5) | 85 (50.6) | 0.001 |
| Semi-urban | 158 | 114 (38.6) | 44 (26.2) |  |
| Rural | 87 | 49 (16.6) | 38 (22.6) |  |
| Level of education, n=460 |  |  |  |  |
| None | 73 | 61 (20.7) | 12 (7.1) | 0.001 |
| Primary | 178 | 121 (41.0) | 57 (33.9) |  |
| Secondary | 136 | 78 (26.4) | 58 (34.5) |  |
| College and above | 73 | 35 (11.9) | 38 (22.6) |  |
| Stroke risk factor |  |  |  |  |
| Hypertension | 336 | 205 (69.5) | 131 (78.0) | 0.05 |
| On regular treatment | 129 | 80 (27.1) | 49 (29.2) | 0.504 |
| Diabetes Mellitus | 67 | 42 (14.2) | 25 (14.9) | 0.644 |
| On regular treatment | 38 | 27 (9.2) | 11 (6.5) | 0.723 |
| Smoking | 3 | 1 (0.3) | 2 (1.2) | 0.285 |
| Alcohol intake | 7 | 4 (1.4) | 3 (1.8) | 0.719 |
| Previous stroke | 53 | 27 (9.2) | 26 (15.5) | 0.043 |
| Cardiac diseases | 18 | 9 (3.1) | 9 (5.4) | 0.226 |
| Pre-morbid mRS, n=462 | |  |  |  |
| No symptoms (score 0) | 272 | 221 (74.9) | 51 (30.4) | 0.001 |
| Able to carry out activities (score 1) | 24 | 9 (3.1) | 15 (8.9) |  |
| Slight disability (score 2) | 37 | 14 (4.8) | 23 (13.7) |  |
| Moderate disability (score 3) | 68 | 24 (8.1) | 44 (26.2) |  |
| Moderate to severe disability (score 4) | 47 | 18 (6.1) | 29 (17.3) |  |
| Severe disability (score 5) | 14 | 9 (3.1) | 5 (3.0) |  |
| Admission stroke severity-NIHSS, n=454 |  |  |  |  |
| Minor scores of 1-4 | 6 | 4 (1.4) | 2 (1.2) | 0.087 |
| Moderate scores of 5-15 | 84 | 45 (15.3) | 39 (23.2) |  |
| Moderate to severe scores of 16-20 | 193 | 132 (44.7) | 61 (36.3) |  |
| Severe scores of 21-42 | 171 | 114 (38.6) | 57 (33.9) |  |
| Discharge mRS, n=429 |  |  |  |  |
| No symptoms (score 0) | 4 | 4 (1.4) | 0 (0.0) | 0.060 |
| No significant disability (score 1) | 11 | 2 (0.7) | 9 (5.4) |  |
| Slight disability (score 2) | 43 | 25 (8.5) | 18 (10.7) |  |
| Moderate disability (score 3) | 154 | 100 (33.9) | 54 (32.1) |  |
| Moderate to severe disability (score 4) | 73 | 57 (19.3) | 16 (9.5) |  |
| Severe disability (score 5) | 7 | 5 (1.7) | 2 (1.2) |  |
| Dead (score 6) | 137 | 94 (31.9) | 43 (25.6) |  |

mRS- modified Rankin Scale, NIHSS- National Institutes of Health Stroke Scale, N-number, IQR- Interquartile range.

**Supplementary Table S2:** Proportion of patients who underwent additional diagnostic vessel imaging.

| Variables | Frequency (N = 295) | Percentages (%) |
| --- | --- | --- |
| CT Angiography (CTA) head and neck | 18 | 6.1 |
| Transthoracic echocardiography (TTE) | 61 | 20.7 |
| Carotid Doppler ultrasound | 14 | 5.6 |

All tests were performed only when clinically indicated, not as routine screening. The low completion rates (CTA 6.1%, TTE 20.7%, Carotid Doppler 5.6%) and selective use of additional imaging reflect financial constraints.
